# Disruption of mitonuclear coadaptation and compensatory evolution after an extreme dietary shift in carnivorous butterflies

**DOI:** 10.1093/molbev/msag221

**Published:** 2026-08-27

**Authors:** Runzhao Fang, Xiao Tian, Dan Liang, Peng Zhang

**Affiliations:** School of Life Sciences, Sun Yat-sen University, Guangzhou 510275, China; School of Life Sciences, Sun Yat-sen University, Guangzhou 510275, China; School of Life Sciences, Sun Yat-sen University, Guangzhou 510275, China; Guangdong Provincial Key Laboratory for Aquatic Economic Animals, Sun Yat-sen University, Guangzhou 510275, China; School of Life Sciences, Sun Yat-sen University, Guangzhou 510275, China; Guangdong Provincial Key Laboratory for Aquatic Economic Animals, Sun Yat-sen University, Guangzhou 510275, China

**Keywords:** mitonuclear coevolution, coadaptation, nuclear compensation, mitochondrial genome

## Abstract

Mitochondrial function depends on tight coordination between mitochondrial and nuclear genomes, which requires long-term coevolution to maintain mitonuclear coadaptation. While mitonuclear incompatibility is typically studied in the context of hybridization, other evolutionary scenarios that may disrupt coadaptation between the two genomes remain less explored. Here, we propose that extreme ecological niche shifts may disrupt mitonuclear coadaptation, which we test in carnivorous Miletinae butterflies with an extreme dietary transition. By generating high-quality genome assemblies, we found that Miletinae exhibit extensive chromosomal rearrangements. Comparative phylogenomic analyses revealed a striking asymmetric mitonuclear evolutionary response: Miletinae exhibit elevated mitochondrial nucleotide substitution rates compared to phytophagous relatives, whereas nuclear rates remain stable. This shift reverses the typical lepidopteran pattern where nuclear rates exceed mitochondrial rates. Interestingly, this mitochondrial acceleration is driven primarily by relaxed purifying selection rather than positive selection. To sustain mitochondrial function, the nuclear genome of Miletinae underwent pervasive, multilayered compensatory evolution. We detected strong signatures of positive selection and accelerated evolution in nuclear genes directly interacting with mitochondrial components across oxidative phosphorylation (OXPHOS) complexes, the mitochondrial translation, and replication and transcription machinery. Furthermore, this nuclear compensatory response extends to systems governing mitochondrial homeostasis, including protein quality control and RNA degradation and stabilization. Our results support a model in which extreme ecological transitions can disrupt ancestral mitonuclear coadaptation and promote the emergence of a new coadapted state through systemic nuclear compensation. This study broadens the conceptual framework of mitonuclear coevolution and highlights its role in facilitating evolutionary persistence after major ecological shifts.

## Introduction

Mitochondria are essential organelles whose functions depend on tight coordination between two physically separate genomes (Martin et al. 2015; Hill 2019). Although the mitochondrial genome retains only a small number of genes in animals (Ray 2014), these genes encode core components of oxidative phosphorylation (OXPHOS) and mitochondrial translation, which must operate in concert with a much larger set of nuclear-encoded proteins, known as mitonuclear genes, imported into the organelle (Lotz et al. 2014). As a result, mitochondrial function relies on pervasive interactions between mitochondrial- and nuclear-encoded gene products, including protein–protein, protein–RNA, and protein–DNA interactions (Diodato et al. 2014; Greber and Ban 2016; Hill 2019). This intimate functional coupling has led to the concept of mitonuclear coevolution, whereby evolutionary change in one genome can impose selective pressure on the other, promoting compensatory or coordinated changes that maintain organelle performance and organismal fitness (Rand et al. 2004; Sloan et al. 2018; Hill 2019; Piccinini et al. 2021; Weaver et al. 2022). Over evolutionary time, such reciprocal adjustment is expected to generate mitonuclear coadaptation, namely, a well-matched combination of mitochondrial and nuclear genotypes that is compatible with the physiological demands of a lineage's ecological niche (Rand et al. 2004; Burton and Barreto 2012; Sloan et al. 2018; Hill 2019).

Despite this long-term coadaptation, the two genomes differ fundamentally in their inherited and evolutionary properties. In animals, the mitochondrial genome is usually maternally inherited (Giles et al. 1980), largely nonrecombining (Ballard and Whitlock 2004), and characterized by a smaller effective population size (Moore 1995) and often higher mutation rate than the nuclear genome (Lynch et al. 2006). These asymmetries make the mitochondrial genome more vulnerable to mutational erosion (Lynch and Blanchard 1998; Neiman and Taylor 2009) and may cause it to respond differently to selection (Havird and Sloan 2016). Although mitonuclear coadaptation is essential for mitochondrial function, it is potentially fragile. A large body of work has shown that hybridization can disrupt coadapted mitonuclear combinations by bringing together mitochondrial and nuclear genomes with distinct evolutionary histories, sometimes resulting in reduced OXPHOS performance, developmental defects, or hybrid breakdown (Ellison and Burton 2006; Niehuis et al. 2008; Burton and Barreto 2012; Barreto and Burton 2013a; Healy and Burton 2023). This hybridization-centered framework has greatly advanced our understanding of mitonuclear incompatibility, but it leaves a broader question unresolved: can mitonuclear coadaptation also be disrupted in the absence of hybridization, through evolutionary change within a lineage itself?

One plausible but little explored scenario is extreme ecological niche shift. Because mitochondrial energy metabolism underpins organismal performance, ecological transitions that substantially alter metabolic substrates, energetic requirements, or physiological demands may impose new selective regimes on mitonuclear function (Hill 2019; Koch et al. 2021). Studies in model organisms such as fruit flies and yeasts have shown that different mitonuclear genotype combinations can markedly affect feeding preferences (Camus and Inwongwan 2023; Dobson et al. 2023), life-history traits (Camus et al. 2020), and organismal fitness (Sujkowski et al. 2019; Nguyen et al. 2020; Rodríguez et al. 2021). These studies suggest that mitonuclear genotype is closely linked to ecological adaptation and that well-adapted lineages are expected to possess mitonuclear genotypes optimized for their ecological niches (Hill et al. 2019; Nguyen et al. 2025). If ecological adaptation depends on optimized mitonuclear genotypes, then dramatic ecological transitions should fundamentally alter the selective landscape acting on mitonuclear interactions. Under such circumstances, mitonuclear genotype adapted to the energetic metabolic regimes of ancestral niches may lose fitness under novel metabolic demands, resulting in relaxed ancestral selective constraints. This phenomenon can be observed in taxa that have undergone successful extreme ecological transitions from their ancestral niches, such as the transition from free-living to parasitic lifestyles in parasitic barnacles (Jung et al. 2025) and the shift from parasitic to predatory lifestyles in mites (Havird et al. 2026). Meanwhile, the strong selective pressures imposed by the new ecological niche may amplify evolutionary differences between mitochondrial and nuclear genomes, leading to asymmetric evolutionary responses and ultimately disrupting preexisting mitonuclear coadaptation. Under this biological scenario, there are two possible evolutionary outcomes of such mitonuclear incompatibility: (i) failure to adapt to the new niche, where reduced mitochondria efficiency influences survival and reproduction (Ellison and Burton 2006; Niehuis et al. 2008); or (ii) restoration of coadaptation through renewed mitonuclear coevolution, whereby nuclear and mitochondrial genomes undergo compensatory or synergistic changes to produce a genotype better suited to the new niche (Barreto and Burton 2013b; Sloan et al. 2017). If so, extreme niche transitions could provide a natural route to mitonuclear mismatch and subsequent compensatory coevolution, even without hybridization.

Lepidoptera provide an attractive research system for testing this possibility. The evolutionary history of butterflies and moths has remained remarkably conservative with respect to trophic ecology. More than 99% of butterflies and moths are phytophagous, indicating that the ancestral lepidopteran mitonuclear system evolved under a plant-based nutritional regime that has characterized the order for over 200 million years (Kawahara et al. 2023; Rubinoff et al. 2025). This long-term trophic conservatism provides a well-defined ancestral background against which the evolutionary consequences of a major dietary transition can be evaluated. Against this background, carnivory is exceptionally rare in Lepidoptera, accounting for less than 0.13% of species, and carnivorous lineages are often evolutionarily short-lived or geographically restricted, seldom persisting or diversifying extensively (Pierce 1995; Rubinoff et al. 2025). The butterfly subfamily Miletinae is therefore an unusual and informative exception. Comprising roughly 190 species distributed widely across Asia and Africa, Miletinae larvae are predominantly carnivorous, feeding mainly on hemipterans (Kaliszewska et al. 2015). Their diversity and broad distribution indicate that this lineage has not only crossed a major trophic boundary but also persisted and diversified after doing so. This makes Miletinae a particularly informative natural system for investigating whether a long-term shift from phytophagy to carnivory is associated with unusual patterns of mitonuclear evolution.

Such a transition may be especially relevant to mitonuclear coadaptation because different diets impose different metabolic inputs and physiological demands. Relative to a carbohydrate-rich phytophagous lifestyle, carnivory entails greater dependence on protein- and lipid-derived metabolism, potentially affecting the mitochondrial energy pathways (Yu et al. 2014; Putti et al. 2015; Aw et al. 2018; Havird et al. 2019). If extreme dietary transition destabilized an ancestral mitonuclear configuration, we would expect to observe signatures of asymmetric evolution between mitochondrial and nuclear genomes, together with evidence that nuclear genes involved in mitochondrial function experienced compensatory or coordinated evolution (Barreto and Burton 2013b; Sloan et al. 2017). Conversely, if coadaptation was maintained without disruption, then Miletinae should show no unusual departure from the mitonuclear evolutionary patterns typical of phytophagous butterflies.

In this study, we test the hypothesis that a drastic ecological niche shift can disrupt mitonuclear coadaptation by using the carnivorous butterfly subfamily Miletinae as a natural evolutionary system. We generated high-quality nuclear genome assemblies for three species and mitochondrial genomes for six species representing the major clades of the subfamily, and compared them with genomic data from closely related phytophagous butterflies. We tested whether the shift to carnivory in Miletinae was accompanied by asymmetric evolutionary responses between the mitochondrial and nuclear genomes, whether the mitochondrial genome experienced adaptive evolution or relaxation of purifying selection, and whether nuclear genes involved in mitochondrial function showed signatures of compensatory evolution. By addressing these questions, our study aims to test whether extreme ecological niche shifts can be accompanied by disruption of mitonuclear coadaptation and to further explore the depth and breadth of mitonuclear coevolution following such disruption.

## Results

### New mitochondrial and nuclear genome resources for Miletinae

**Figure 1:**
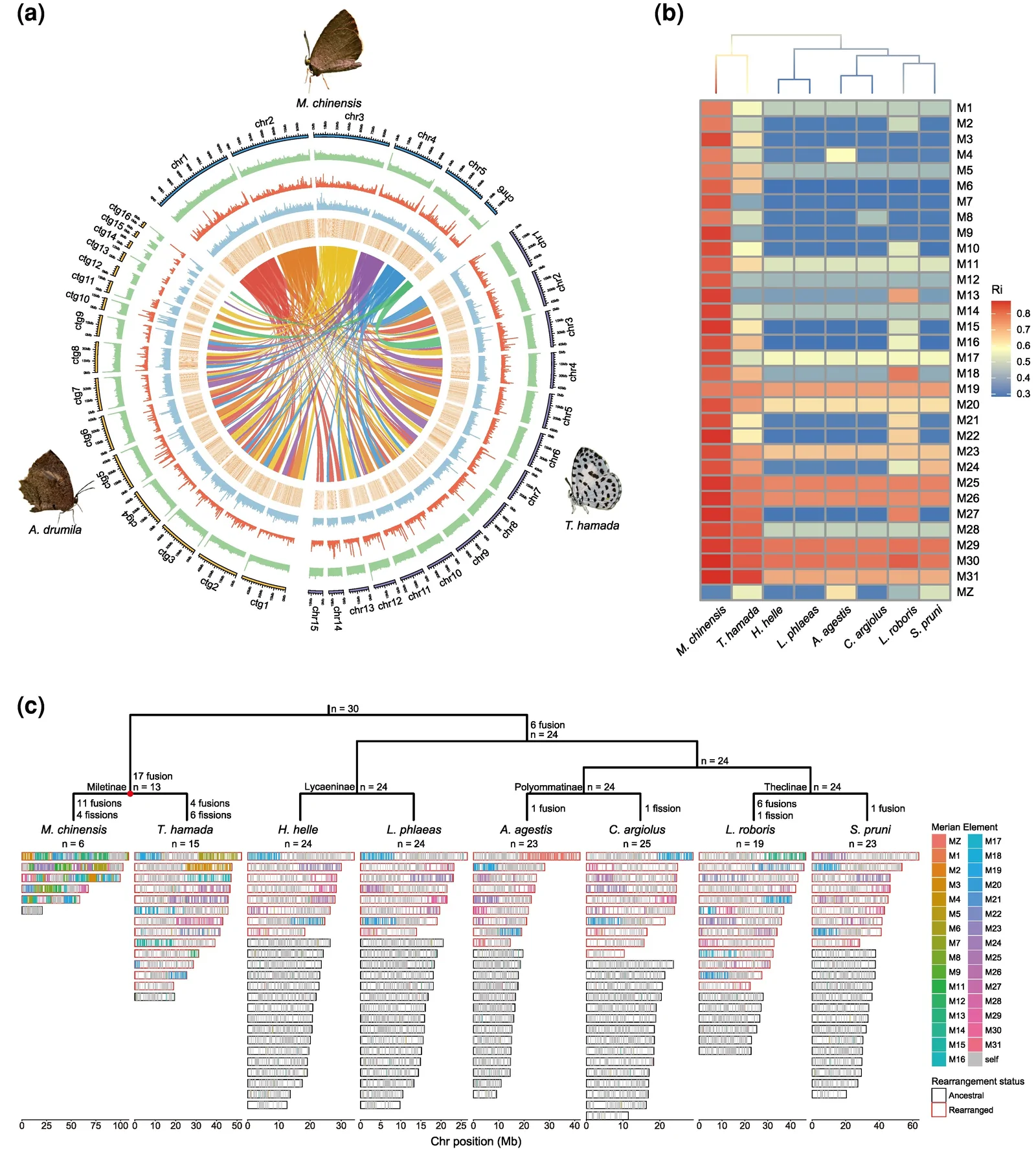
Genome assemblies and extensive chromosomal rearrangements in Miletinae. a) Circos plots showing the genomic features of *A. drumila*, *M. chinensis*, and *T. hamada*. The central links depict syntenic blocks between *M. chinensis* and the other two species. From the inner to the outer rings, the tracks represent gene density, transposable element abundance, LTR abundance, GC content, and the lengths of assembled chromosomes or contigs. b) Chromosomal rearrangement patterns across lycaenid species. Phylogenetic branches were color-coded according to the genome-wide rearrangement index (Ri). The heatmap shows Ri values for each ALG (Merian element) in lycaenid species, with color intensity ranging from 0 (no rearrangement) to 1 (maximum rearrangement). c) Karyotype evolution and Merian element dynamics in lycaenid species. The phylogeny is annotated with inferred chromosomal fusion and fission events and haploid chromosome numbers (*n*) at each node. Chromosomes are shown as rectangles. Orthologues retained within their ancestral Merian elements are shown in gray, whereas orthologues derived from other Merian elements are shown in color. Chromosomes involved in inferred fusion and/or fission events are highlighted in red.

We sampled six species of Miletinae (*Taraka hamada*, *Taraka shiloi*, *Miletus chinensis*, *Miletus mallus*, *Allotinus drumila*, and *Spalgis epius*) for mitochondrial genome sequencing, covering three major clades of the subfamily (Kaliszewska et al. 2015). Due to DNA quality limitation, only *T. hamada*, *M. chinensis*, and *A. drumila* were used for whole-genome sequencing. An initial genome survey of *T. hamada* estimated a genome size of approximately 456.66 Mb and a low heterozygosity rate of 0.58% (Figure S1). We then generated a total of 62.60 Gb of PacBio HiFi reads for the three species (∼40× coverage per species) for de novo genome assembly (Table S1). The resulting assemblies ranged from 476.71 to 569.93 Mb, with contig N50 values between 18.20 and 99.76 Mb (Table S2). The *A. drumila* genome assembly contained 16 major contigs, accounting for 98.20% of the genome, and the assemblies of *M. chinensis* and *T. hamada* were further scaffolded with Hi-C data into 6 and 15 pseudo-chromosomes, respectively (Fig. 1a; Figure S2). All three assemblies were highly complete, with BUSCO scores of 98.00% to 98.30%. Notably, *M. chinensis* contained six fully gap-free telomere-to-telomere chromosomes, representing the lowest chromosome number yet reported in Lepidoptera (Figure S3). 15,326, 15,650, and 13,793 protein-coding genes were annotated in *A. drumila*, *M. chinensis*, and *T. hamada*, respectively, and repetitive elements accounted for 33.75% to 54.03% of the assembled genomes, with LINEs as the dominant repeat class (Tables S3 and S4).

In addition, genome synteny analyses across eight chromosome-level lycaenid genomes revealed extensive disruption of ancestral chromosome organization in the sampled Miletinae species, *M. chinensis* and *T. hamada* (Figure S4). This pattern was quantified using the rearrangement index (Ri) across the 32 ancestral lepidopteran linkage groups (Merian elements), which showed that the two Miletinae genomes had the highest genome-wide rearrangement levels among all sampled lycaenids (Fig. 1b; Table S5) (Lewin et al. 2024; Wright et al. 2024). Nearly all Merian elements exhibited strong rearrangement signals, indicating that chromosome restructuring in Miletinae is a genome-wide property rather than a localized feature of a few chromosomes.

Ancestral reconstruction further showed that this derived genomic architecture was shaped primarily by chromosome fusion. Whereas the last common ancestor of Lycaenidae was inferred to have had a haploid chromosome number of *n* = 30, the ancestral Miletinae lineage experienced 17 fusion events, reducing chromosome number to *n* = 13 (Fig. 1c). Additional lineage-specific rearrangements subsequently remodeled the genomes of *M. chinensis* and *T. hamada*, leaving only a single unrearranged Merian element in each species (MZ in *M. chinensis* and M13 in *T. hamada*; Table S6). In contrast, the other sampled lycaenid subfamilies retained substantially more conservative chromosome organization after only six ancestral fusion events (Fig. 1c). Together, these new data establish a robust genomic foundation for comparative analyses of genome evolution and mitonuclear evolution in Miletinae butterflies, and show that this lineage evolved against a profoundly restructured genomic background.

### Miletinae show asymmetric evolutionary responses between mitochondrial and nuclear genomes

To compare patterns of mitochondrial and nuclear genome evolution between carnivorous Miletinae and phytophagous butterflies, we assembled a dataset of 45 butterfly species representing 7 families and 45 genera, together with 3 non-butterfly lepidopteran species designated as outgroups (Table S7). Because many species lacked annotated nuclear gene sets, we generated protein-coding gene annotations for 34 taxa using the same pipeline applied to the 3 newly assembled Miletinae genomes. We then reconstructed two concatenation-based maximum likelihood (ML) phylogenies, one from 5,150 nuclear BUSCO (nuBUSCO) genes and the other from 13 mitochondrial protein-coding genes (mtPCGs). Because the nuBUSCO phylogeny was more congruent with the accepted butterfly phylogeny than the unconstrained mitochondrial topology (Kawahara et al. 2023), mitochondrial branch lengths were re-estimated under the topology of the nuBUSCO phylogeny to enable direct comparison between the two genomes.

**Figure 2:**
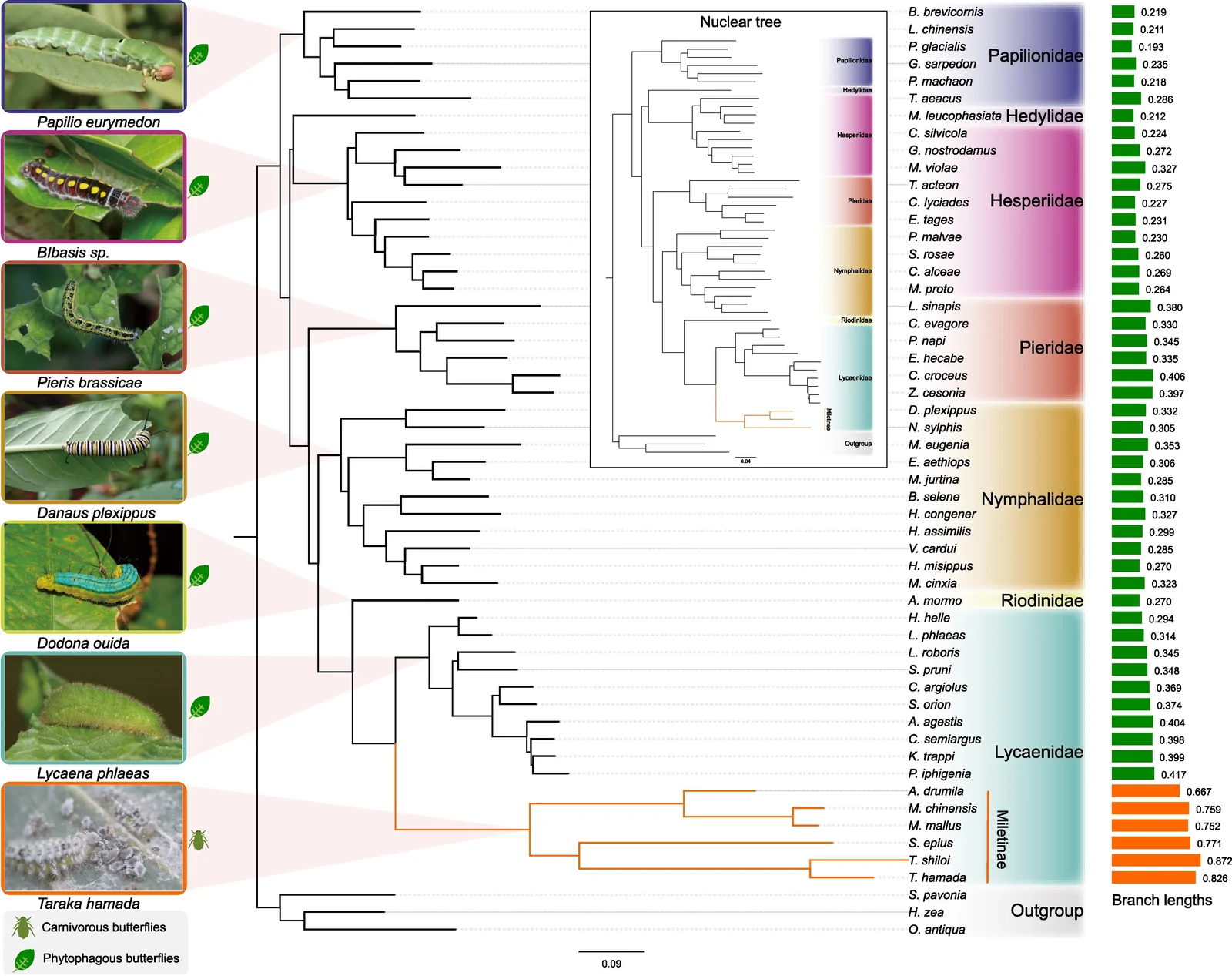
Accelerated mitochondrial evolution in carnivorous Miletinae butterflies. The inset shows the concatenation-based maximum likelihood phylogeny inferred from 5,150 nuclear BUSCO (nuBUSCO) genes under the LG + G4 model. The main phylogeny was estimated from 13 mtPCGs under the mtInv + F + G4 model, with branch lengths re-estimated under the topology constrained to the nuBUSCO tree. Carnivorous Miletinae lineages are highlighted in orange in both phylogenies. The bar plot on the right shows mitochondrial root-to-tip branch lengths for each species, revealing substantially longer mitochondrial branches in Miletinae than in phytophagous butterflies. Representative larval photographs are shown for each family or subfamily to illustrate feeding habits, with green leaf icons indicating phytophagy and prey-associated icons indicating carnivory. Photographs were kindly provided by Marc Kummel, John Horstman, Marcus F. C. Ng, Dean Morley, and Scot Nelson.

This comparison revealed a striking contrast between mitochondrial and nuclear evolutionary patterns in Miletinae and phytophagous butterflies. The mean root-to-tip branch length of Miletinae species in the mtPCGs phylogeny was approximately 2.55-fold longer than that observed in phytophagous butterflies; by contrast, the mean root-to-tip branch lengths were broadly comparable between Miletinae and other butterflies in the nuBUSCO phylogeny (Fig. 2). Consistent with this pattern, the mean nucleotide substitution rate of mtPCGs was significantly higher in Miletinae (5.8 × 10^−3^ substitutions/site/Myr) than in phytophagous butterflies (2.0 × 10^−3^ substitutions/site/Myr; Mann–Whitney *U* test, *P* = 1.5 × 10^−7^; Figure S5a). In contrast, nucleotide substitution rates estimated from nuBUSCO genes did not differ significantly between the two groups (Figure S5b; *P* = 0.40).

This shift also altered the usual balance between mitochondrial and nuclear rates within butterflies. Across phytophagous butterflies, nuclear coding DNA sequence (CDS) substitution rates were generally higher than mitochondrial rates (Figure S5c; *P* = 2.3 × 10^−14^), consistent with the typical mitonuclear evolutionary pattern observed in Lepidoptera (Tao et al. 2024). In Miletinae, however, this relationship was reversed: the mitochondrial genome evolved faster than the nuclear genome (Figure S5d; *P* = 4.8 × 10^−2^). Together, these results indicate that the transition to carnivory in Miletinae was accompanied by a pronounced acceleration of mitochondrial, but not nuclear, sequence evolution, resulting in strongly asymmetric evolutionary responses between the two genomes.

Because carnivorous Miletinae originated from phytophagous ancestors, we tested whether the association between diet and mitochondrial substitution rate remained significant after accounting for phylogenetic relatedness. Phylogenetic generalized least squares (PGLS) analysis showed that carnivory was still significantly associated with elevated mitochondrial substitution rates after controlling for shared ancestry (β = 0.00403 ± 0.00099, *t* = 4.08, *P* = 2.0 × 10^−4^). We further asked whether this rate elevation was associated with the dietary transition itself or with the subsequent evolution of carnivorous lineages. Branch-specific rate analysis identified the branch leading to carnivorous Miletinae as a significant acceleration branch in the rate-shift test, with an empirical probability falling in the upper tail of the background distribution (Figure S6; probability = 0.99). In addition, branch-specific rate-change estimates showed that the magnitude of acceleration on this transition branch was significantly greater than that observed on descendant carnivorous branches (permutation test, *P* = 1.9 × 10^−2^). These results suggest that the elevated mitochondrial rate in Miletinae arose specifically during the initial dietary transition to carnivory, rather than through a uniform or continuous rate increase across carnivorous lineages after the diversification of Miletinae.

### Accelerated mitochondrial evolution in Miletinae is genome-wide and primarily driven by relaxed purifying selection

**Figure 3:**
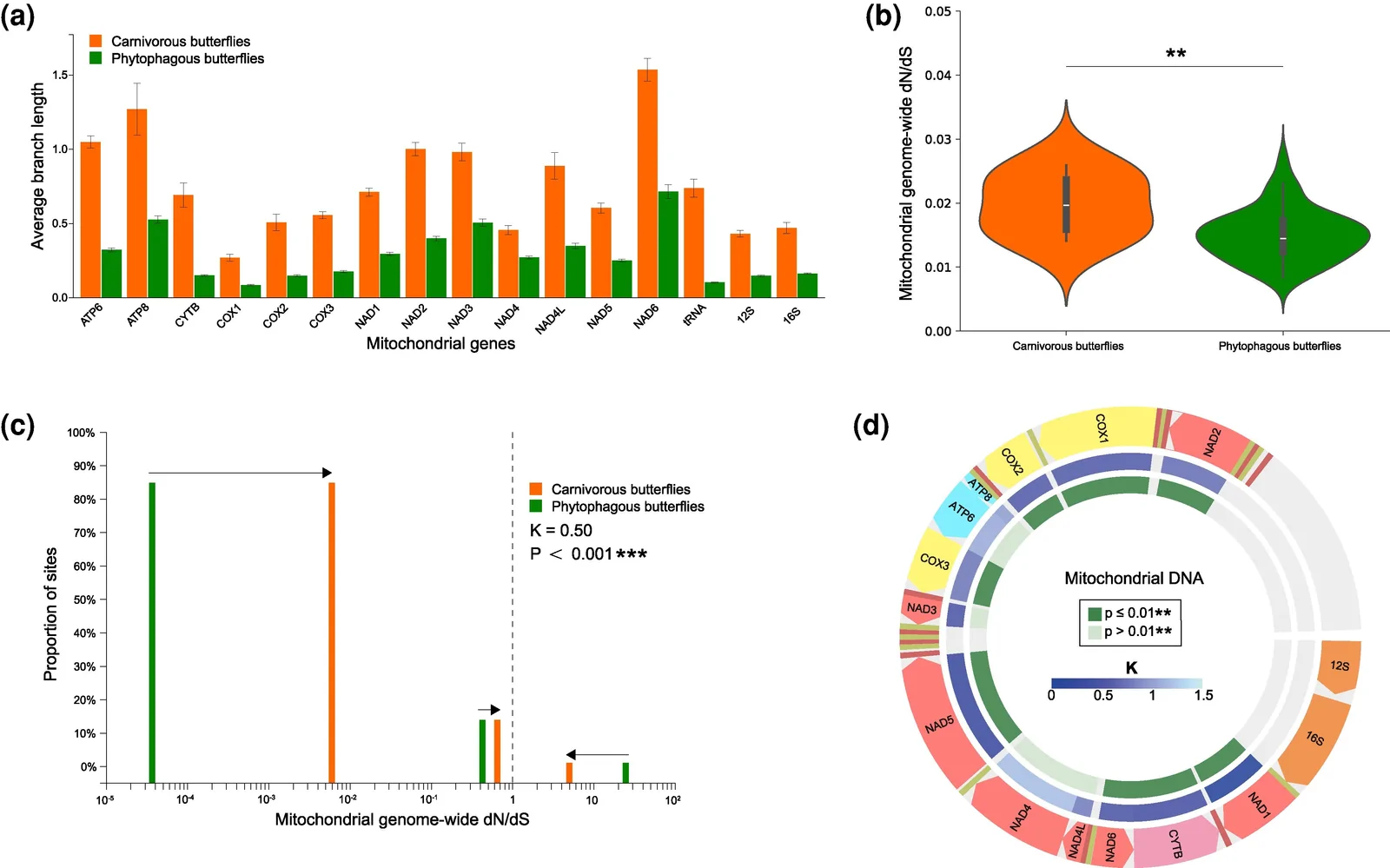
Evolutionary characteristics of the mitochondrial genome in Miletinae. a) Comparison of average branch lengths for individual mitochondrial genes between Miletinae and phytophagous butterflies. The branch length for mitochondrial tRNAs was estimated from a concatenated alignment of 22 tRNA genes. Error bars represent standard errors. b) Comparison of genome-wide mitochondrial *dN/dS* ratios between Miletinae and phytophagous butterflies. Statistical significance was assessed using the Mann–Whitney *U* test. c) Distribution of selection categories across mitochondrial genomic sites inferred using RELAX. Bars represent the proportions of sites assigned to purifying, neutral, and positive selection in Miletinae and phytophagous butterflies. Statistical significance was assessed using LRTs. d) Circos plot showing the intensity of relaxed selection across the 13 mtPCGs of Miletinae. From the inner to the outer rings: *P*-values, selection intensity parameter (*K*), and gene names. In all panels, asterisks indicate significance levels: ns = not significant; * < 0.05; ** < 0.01; *** < 0.001.

To test whether the elevated mitochondrial evolutionary rate in Miletinae was confined to protein-coding genes or instead reflected a genome-wide pattern, we reconstructed phylogenetic trees for each mitochondrial gene and used mean branch length as a proxy for evolutionary rate. All mitochondrial genes, including protein-coding genes, rRNAs, and tRNAs, showed longer branches in carnivorous Miletinae than in phytophagous butterflies (Fig. 3a), indicating that the rate increase extends across the entire mitochondrial genome rather than being restricted to mtPCGs.

To investigate the selective pressures causing this genome-wide rate elevation, we estimated mitochondrial genome-wide *dN/dS* ratios across the butterfly phylogeny using CodeML (Yang 2007). Miletinae showed significantly higher genome-wide *dN/dS* values than phytophagous relatives (Mann–Whitney *U* test, *P* = 9.6 × 10^−3^), pointing to a shift in selective regime (Fig. 3b). Because elevated *dN/dS* can arise either from positive selection or from relaxed purifying selection, we next used RELAX model (Wertheim et al. 2015) to distinguish between these alternatives. RELAX classifies sites into purifying, neutral, and positively selected categories and estimates a selection intensity parameter (*K*), with *K* < 1 indicating relaxed selection and *K* > 1 indicating intensified selection. In Miletinae, all three site classes shifted toward greater neutrality relative to phytophagous butterflies, consistent with an overall relaxation of selective constraint. Accordingly, the elevated *dN/dS* in Miletinae is best explained by relaxed purifying selection rather than adaptive evolution (Fig. 3c; *K* = 0.5; likelihood ratio test [LRT], *P* = 2.7 × 10^−34^).

Although purifying selection appears to be broadly relaxed across the mitochondrial genome, individual genes could still experience positive selection, and some sites might remain targets of positive selection. We therefore applied RELAX and branch-site models to each of the 13 mtPCGs. Eight genes showed significant evidence of relaxed selection (*K* < 1 and *P* < 0.01), whereas none showed evidence of positive selection (Fig. 3d). Taken together, the genome-wide and gene-level analyses consistently indicated that the elevated mitochondrial evolutionary rate in Miletinae was driven primarily by relaxed purifying selection rather than adaptive evolution.

### Nuclear genes associated with mitochondrial function show pervasive multilayered signatures of compensatory evolution

Given the unusually accelerated yet apparently largely nonadaptive mitochondrial evolution in Miletinae, we next asked whether the nuclear genome shows signatures of compensatory evolution in genes associated with mitochondrial function. To address this question, we conducted comparative analyses across 20 butterfly species and examined nuclear evolution at 3 levels: gene content, coding sequences, and regulatory elements.

**Figure 4:**
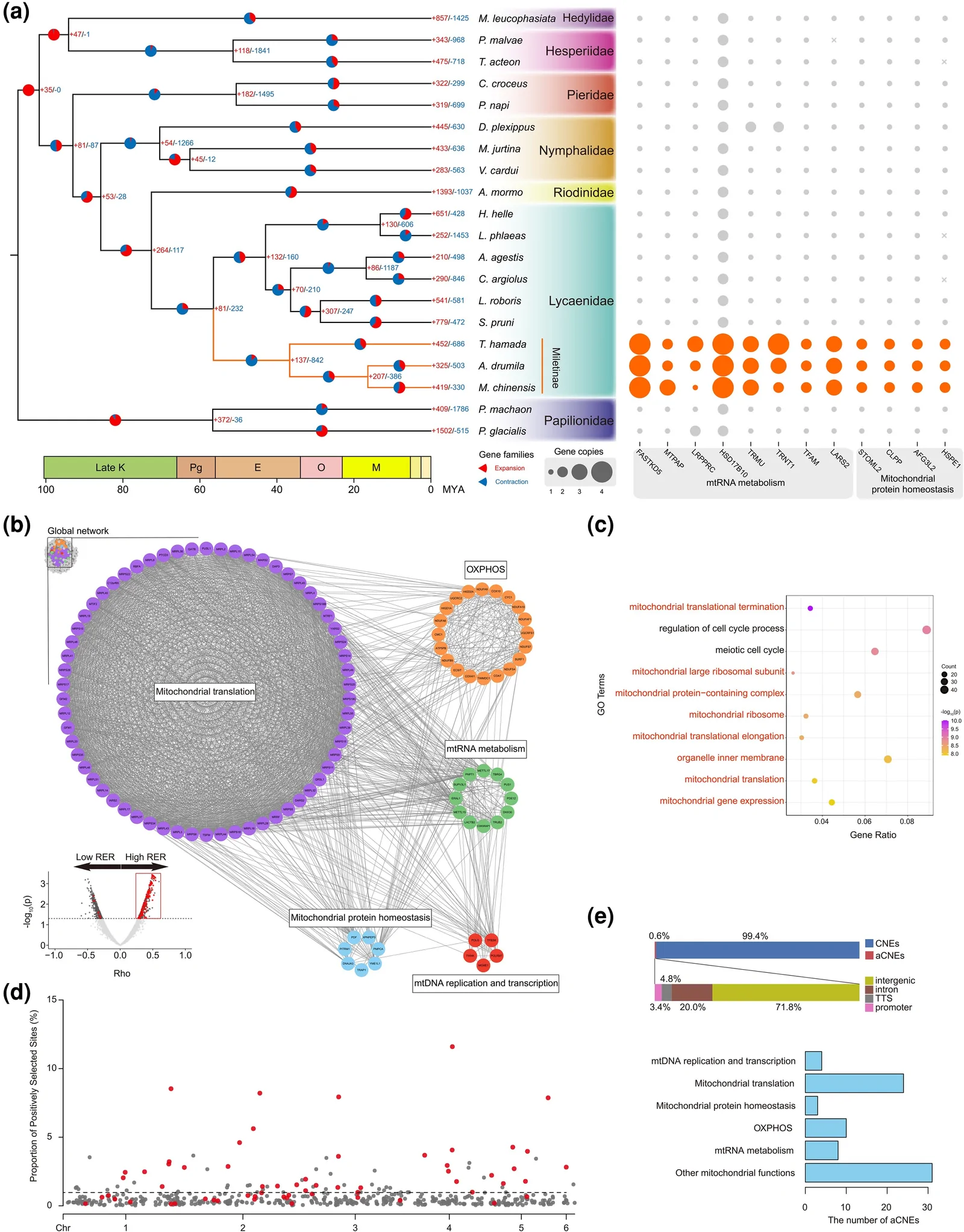
Multilayered nuclear compensatory evolution in Miletinae. a) Gene family evolution across the 20 butterfly species used for comparative genomic analyses. The number of gene family expansions and contractions is shown along branches in red and blue, respectively. Pie charts represent the proportions of expanded and contracted gene families. The bubble plot on the right shows copy-number variation of expanded mitonuclear genes in each species, grouped by mitochondrial functional category. b) Protein RER analysis and protein–protein interaction network of HRERGs in Miletinae. The inset scatter plot shows genes with elevated or reduced RER on the Miletinae branch (red dots refer to mitonuclear genes). The network shows interactions among HRERGs, with mitonuclear genes grouped into major functional clusters. c) The top 10 significantly enriched gene ontology (GO) terms for PSGs were identified on the ancestral branch of Miletinae. d) Manhattan plot showing all PSGs and their selection intensities (red dots refer to mitonuclear genes). The x-axis denotes chromosomal positions, and the y-axis represents the proportion of positively selected sites relative to protein length. e) CNE evolution in Miletinae. The upper stacked bar plot shows the proportions of CNEs and Miletinae-specific aCNEs. The middle stacked bar plot shows the genomic distribution of aCNEs. The lower bar plot shows the number of mitonuclear-associated aCNEs assigned to different mitochondrial functional pathways.

At the level of gene content, analysis of gene family evolution along the ancestral branch of Miletinae identified 137 significantly expanded gene families (Fig. 4a; Table S8), including 12 associated with mitochondrial RNA metabolism and mitochondrial protein homeostasis. Most of these gene families are single-copy in other phytophagous butterflies, indicating that their expansion is lineage-specific to Miletinae (Fig. 4a). Although these genes are not structural subunits of OXPHOS complexes or the mitoribosome, they participate in core mitochondrial processes such as precursor RNA processing, RNA modification, and protein quality control (Quirós et al. 2015; D'Souza and Minczuk 2018; Santonoceto et al. 2024), suggesting broader remodeling of the nuclear machinery that supports mitochondrial function.

Coding sequence analyses revealed a similar pattern. Relative evolutionary rate (RER) analyses identified 334 high RER genes (HRERGs) on the Miletinae lineage (Fig. 4b; Table S9), of which 136 were mitonuclear genes, corresponding to a 5.5-fold enrichment over random expectation (Figure S7a; hypergeometric test, *P* = 6.7 × 10^−70^). These genes were enriched in multiple core mitochondrial functions (Figure S8; Table S10), and protein–protein interaction analyses grouped them into 5 major functional clusters, with mitochondrial translation having the largest number of genes, followed by OXPHOS (Fig. 4b). Branch-site analyses further identified 495 positively selected genes (PSGs) on the ancestral branch of Miletinae (Table S11), including 61 mitonuclear genes, again representing a significant enrichment over random expectation (Figure S7b; *P* = 5.1 × 10^−5^). GO enrichment analysis on these PSGs showed that eight of the top ten significantly enriched GO terms were associated with mitochondrial functions (Fig. 4c; Table S12). Moreover, the top ten PSGs ranked by selection intensity were all mitochondria-associated (Fig. 4d; Table S11), indicating that adaptive signals were predominantly concentrated in mitochondrial-related biological processes.

Regulatory element evolution exhibited this pattern as well, although less strongly. Whole-genome alignments of 20 butterfly species identified 247,860 conserved noncoding elements, among which 1,522 were inferred to be Miletinae-specific accelerated conserved noncoding elements (aCNEs) (Fig. 4e; Table S13). These aCNEs were located predominantly in intergenic regions, and their associated genes were mainly enriched for functions related to gene regulation and development, while categories related to mitochondrion localization and respiratory chain complex assembly were also present (Table S14). Among these aCNEs, 80 were linked to mitonuclear genes, but this association was not significantly enriched (Figure S7c; hypergeometric test, *P* = 0.72). Assignment of these mitonuclear-associated aCNEs to specific pathways showed that many were related to mitochondrial translation, whereas others were associated with a broader range of mitochondrial functions, including amino acid metabolism (Fig. 4e; Table S13). Thus, regulatory element changes may have made a more limited contribution to nuclear compensatory evolution in Miletinae.

Taken together, these results show that nuclear evolution in Miletinae was remodeled at multiple levels in association with altered mitochondrial evolution. This multilayered pattern suggests that nuclear responses in this lineage are not random or diffuse, but instead are preferentially concentrated in functions most likely to compensate for the altered mitochondrial genome.

### Compensatory evolution is concentrated in nuclear genes directly interacting with mitochondrial components

#### OXPHOS complexes

**Figure 5:**
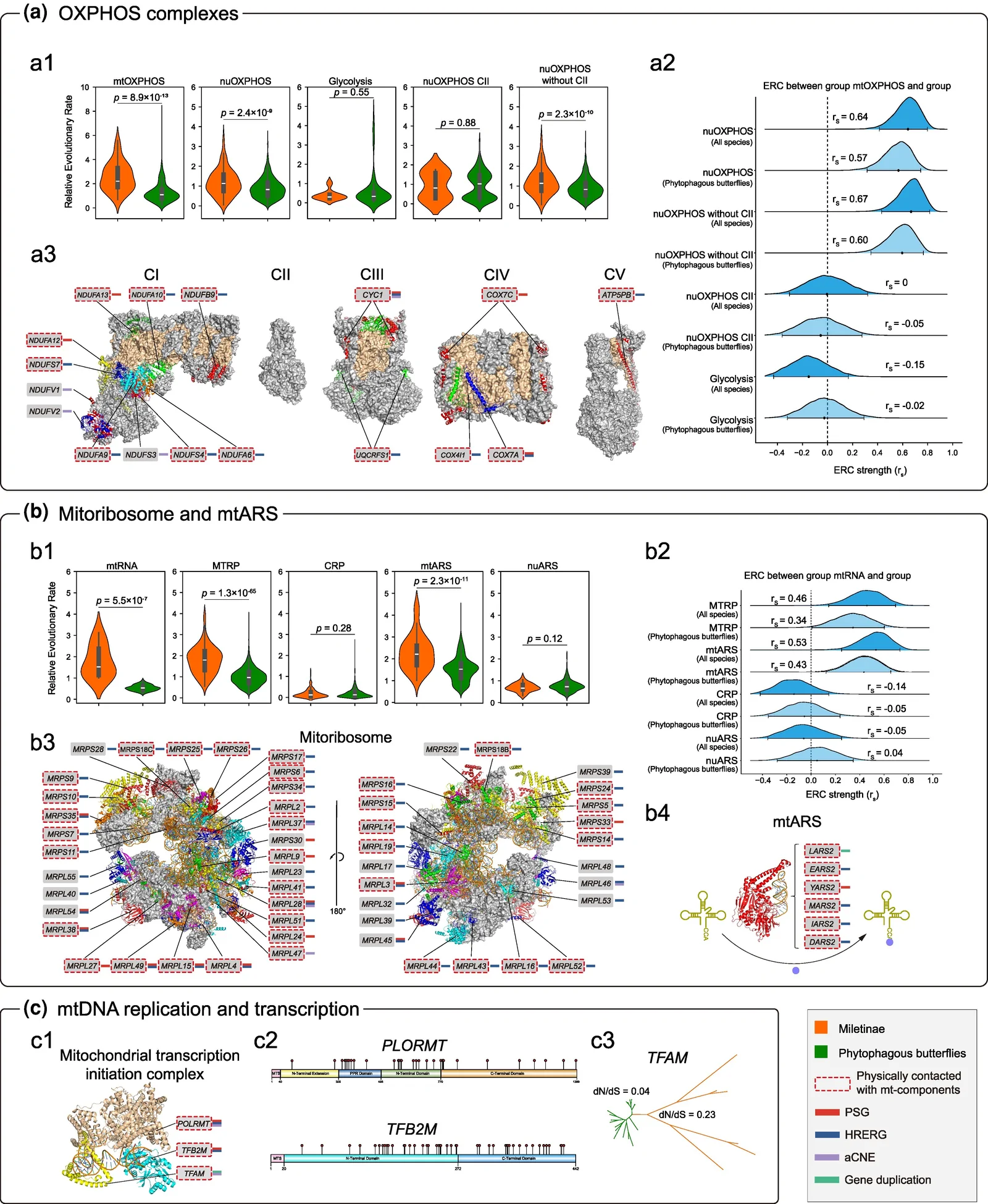
Compensatory evolution at direct mitonuclear interaction interfaces in Miletinae. a) OXPHOS complexes: a1) Comparisons of RERs among different gene groups between Miletinae and phytophagous butterflies. a2) ERC between the mtOXPHOS supergene matrix and the supergene matrices of different nuclear gene groups. a3) Structures of OXPHOS complexes. b) Mitoribosome and mtARS: b1) RER comparisons across gene groups between Miletinae and phytophagous butterflies. b2) ERC between the mtRNA supergene matrix and supergene matrices of different nuclear gene groups. b3 and b4) Structures of the mitoribosome and a representative mtARS. c) mtDNA replication and transcription: c1) Structure of the mitochondrial transcription initiation complex. c2) Domain schematics of *POLRMT* and *TFB2M*, with positively selected sites marked along the protein sequences. c3) Phylogeny of the *TFAM* gene family, with Miletinae branches highlighted in orange. In all structural panels, mitochondrial components are shown as wheat-colored surfaces, selected nuclear-encoded proteins as colored cartoons, and other components as gray surfaces. The red dashed boxes highlight nuclear proteins that have direct physical contact with mitochondrial components.

Among the nuclear systems associated with mitochondrial function, the clearest compensatory signals in Miletinae were concentrated in the OXPHOS complexes, representing the protein–protein interaction between mitochondrial- and nuclear-encoded gene products (Hill 2019). Consistent with the elevated rate of mitochondrial evolution in this lineage, mtOXPHOS genes showed significantly higher RER in Miletinae than in phytophagous butterfly species (Fig. 5a1; Mann–Whitney *U* test, *P* = 8.9 × 10^−13^). Correspondingly, their nuclear counterparts, the nuOXPHOS genes, exhibited the same elevated pattern (*P* = 2.4 × 10^−9^), whereas glycolysis genes, another energy-producing pathway that is entirely nuclear-encoded, showed no significant rate difference between carnivorous and phytophagous lineages (*P* = 0.55). The same contrast was also evident within OXPHOS itself: complex II, which is encoded entirely by the nuclear genome, showed no elevation in RER (*P* = 0.88), whereas the signal for accelerated evolution was significant for nuOXPHOS genes when complex II was excluded (Fig. 5a1; *P* = 2.3 × 10^−10^).

Evolutionary rate covariation (ERC) analyses further supported this conclusion. The RERs of mtOXPHOS and nuOXPHOS genes were strongly positively correlated across all butterfly species (*r_s_* = 0.64), whereas no significant correlation was detected between mtOXPHOS and either glycolysis genes (*r_s_* = 0) or complex II (*r_s_* = −0.15) (Fig. 5a2). When Miletinae were excluded, the positive correlation between mtOXPHOS and nuOXPHOS persisted but was somewhat weaker (*r_s_* = 0.57), suggesting that Miletinae modestly strengthen a broader mitonuclear coevolutionary signal across butterflies (Fig. 5a2). Structural information provided an additional layer of support. Among the 17 nuOXPHOS genes showing signatures of compensatory evolution in Miletinae, 14 exhibited compensatory sequence changes, as reflected by elevated RER or positive selection, and all 14 encode proteins that directly interact with mtOXPHOS subunits (Fig. 5a3). By contrast, the remaining three genes, *NDUFV1*, *NDUFV2*, and *NDUFS3*, do not directly contact mitochondrial-encoded partners, showed no compensatory sequence changes, only possessed aCNEs in their nearby regions (Fig. 5a3). Together, these results show that compensatory change in the OXPHOS system is concentrated primarily in nuclear subunits that directly interact with mitochondrial proteins.

#### Mitoribosome and mtARS

A similarly strong pattern was also observed in the mitochondrial translation system, which represents a second class of direct mitonuclear coupling through protein–RNA interactions (Hill 2019). mtRNA genes (rRNA and tRNA) showed significantly higher RER in Miletinae than in phytophagous butterfly species (Fig. 5b1; *P* = 5.5 × 10^−7^). Correspondingly, both mitoribosomal proteins (MTRP) and mitochondrial aminoacyl-tRNA synthetases (mtARS) showed significantly elevated RER in Miletinae relative to phytophagous butterflies (*P* = 1.3 × 10^−65^ and *P* = 2.3 × 10^−11^). In contrast, the corresponding cytoplasmic systems, cytoplasmic ribosomal proteins (CRP; *P* = 0.28) and cytoplasmic aminoacyl-tRNA synthetases (nuARS; *P* = 0.12), showed no comparable acceleration (Fig. 5b1). Thus, the signal is not a general property of all translational machinery, but is concentrated specifically in the components that interact with mtRNA and support mitochondrial protein synthesis.

ERC analyses again revealed coordinated evolution between mitochondrial and nuclear components. Coevolutionary signals were detected between mtRNA and both MTRP and mtARS (*r_s_* = 0.46 and *r_s_* = 0.53), whereas no significant correlation was observed with their cytoplasmic analogs CRP and nuARS (*r_s_* = −0.14 and *r_s_* = −0.05) (Fig. 5b2). These correlations remained positive, albeit somewhat weaker, when Miletinae were excluded (*r_s_* = 0.34 and *r_s_* = 0.43), suggesting that the carnivorous lineage amplifies an otherwise more modest background pattern of coevolution (Fig. 5b2). Structural information was also consistent with direct compensation. Fifty-three MTRP genes showed signatures of selection in Miletinae, and 38 of them encode proteins that directly contact mitochondrial rRNA (Fig. 5b3). Among mtARS genes, six strictly mitochondrial-localized loci showed lineage-specific selection signals: four (*EARS2*, *MARS2*, *IARS2*, and *DARS2*) were HRERGs, *YARS2* was a PSG, and *LARS2* showed Miletinae-specific expansion (Fig. 5b4). Together, these results show that compensatory change in Miletinae also extends to the mitochondrial translation machinery and is strongest in nuclear genes that directly interact with mitochondrial RNA products.

#### mtDNA replication and transcription

The mitochondrial replication and transcription machinery provided a third, complementary example of direct mitonuclear coupling, here mediated through protein–DNA interactions (Hill 2019). Compared with phytophagous butterflies, carnivorous Miletinae possess significantly larger mitochondrial genomes and expanded control regions (Figure S9; *P* = 1.1 × 10^−3^), indicating that the noncoding regulatory portion of the mitochondrial genome has also undergone substantial evolutionary change. Because the mitochondrial control region contains key regulatory elements involved in both transcription initiation and replication control (Tan et al. 2024), these changes raise the expectation that compensatory evolution may also affect the nuclear factors that recognize and regulate mtDNA.

Consistent with this expectation, several core nuclear factors involved in mitochondrial transcription and replication showed strong signatures of compensatory evolution, most notably *POLRMT*, *TFB2M*, and *TFAM* (Fig. 5c1). *POLRMT*, the mitochondrial RNA polymerase responsible for mtDNA transcription and for generating RNA primers used in replication (Gustafsson et al. 2016), and *TFB2M*, which cooperates with *POLRMT* during transcription initiation by promoting separation of the nontemplate and template strands (Hillen et al. 2017), were both identified as HRERGs and PSGs. In addition, an aCNE was detected near the *POLRMT* locus (Fig. 5c1). Notably, both proteins contained a large number of positively selected sites, and these sites were concentrated mainly in their N-terminal regions (Fig. 5c2), the parts responsible for DNA interaction. This pattern is consistent with adaptive responses to evolutionary changes in the mitochondrial regulatory region in Miletinae (Gustafsson et al. 2016; Hillen et al. 2017).

*TFAM*, a central regulator of mitochondrial DNA packaging, transcription initiation, and replication-associated control-region function (Kozhukhar and Alexeyev 2023), underwent lineage-specific duplication in Miletinae, and aCNEs were also detected in its nearby regions. Moreover, the two Miletinae *TFAM* copies exhibited substantially elevated *dN/dS* values relative to the single-copy *TFAM* of phytophagous butterflies, suggesting functional divergence of Miletinae *TFAM* (Fig. 5c3). Taken together, these coordinated changes in *POLRMT*, *TFB2M*, and *TFAM* indicate that compensatory evolution in Miletinae was not restricted to protein–protein and RNA–protein interfaces, but also extended to the protein–DNA interactions that govern mtDNA transcription and replication.

### Compensatory evolution extends to mitochondrial protein and RNA homeostasis

#### Mitochondrial protein quality control

**Figure 6:**
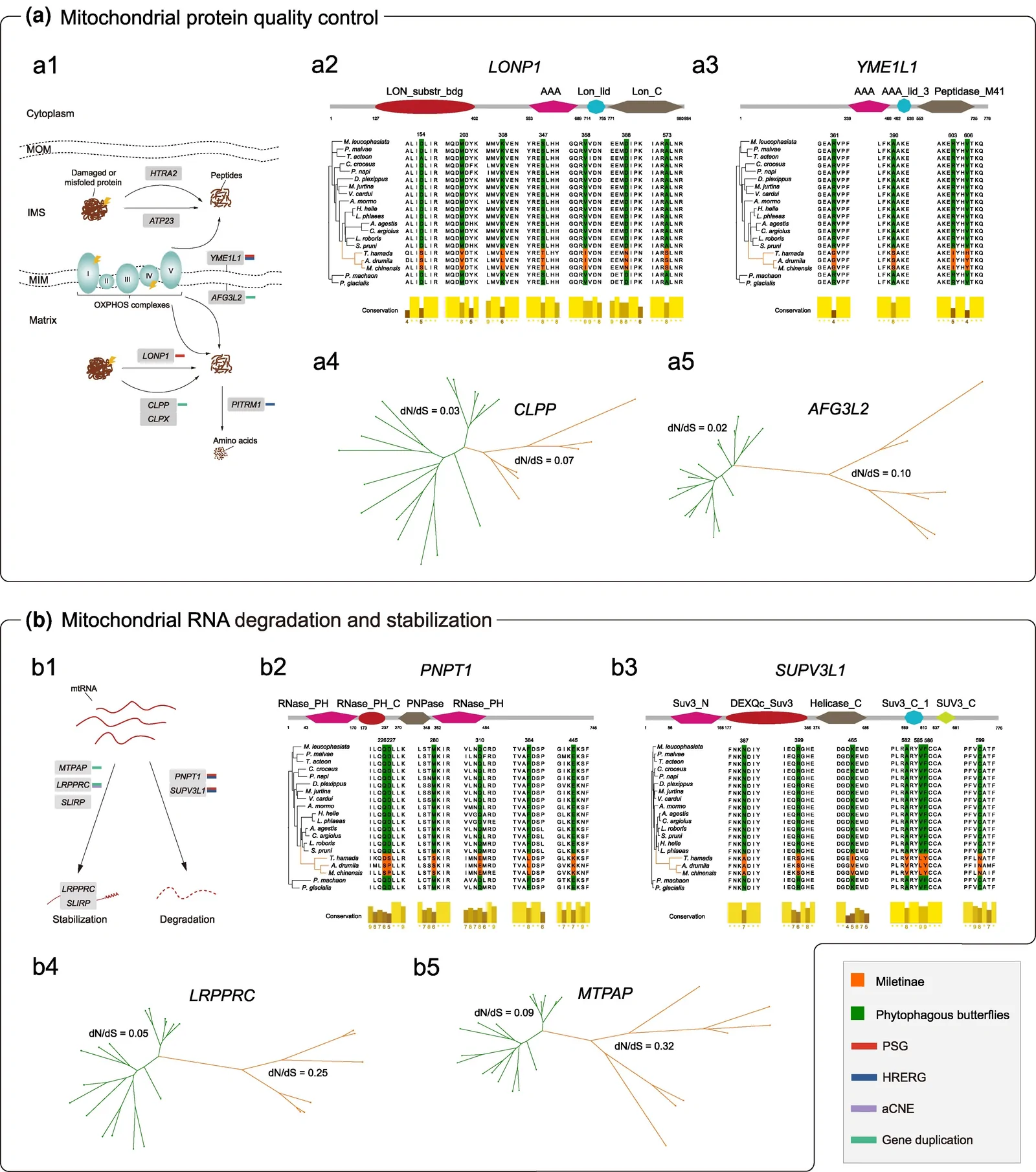
Compensatory evolution in mitochondrial maintenance and homeostasis systems in Miletinae. a) Mitochondrial protein quality control system: a1) Schematic representation of mitochondrial protein quality control, adapted from Quirós et al. (2015). a2 and a3) Gene trees, protein domain architectures, and amino acid alignments for *LONP1* and *YME1L1*. Miletinae-specific amino acid substitutions located within functional domains are highlighted in orange. a4 and a5) Gene family phylogenies of *CLPP* and *AFG3L2*, showing lineage-specific duplications and elevated sequence divergence in Miletinae. b) Mitochondrial RNA degradation and stabilization systems: b1) Schematic representation of mitochondrial RNA degradation and stabilization, adapted from Santonoceto et al. (2024). b2 and b3) Gene trees, protein domain architectures, and amino acid alignments for *PNPT1* and *SUPV3L1*. Miletinae-specific amino acid substitutions within functional domains are highlighted in orange. b4 and b5) Gene family phylogenies of *LRPPRC* and *MTPAP*, showing lineage-specific duplications and elevated sequence divergence in Miletinae.

Beyond the directly coupled mitonuclear systems, the evolutionary response of the nuclear genome extends to systems that maintain mitochondrial macromolecular integrity. One such system is mitochondrial protein quality control, which removes misfolded, oxidized, or improperly assembled proteins and thereby contributes to mitochondrial proteostasis (Quirós et al. 2015). Among the 7 major proteases that constitute this system (*YME1L1*, *AFG3L2*, *LONP1*, and *CLPP*, *ATP23* and *HTRA2*, *PITRM1*), 5 showed clear signatures of lineage-specific evolution in Miletinae (Fig. 6a1). *LONP1*, a matrix-localized AAA+ protease with proteolytic, chaperone, and mtDNA-binding activities (Tang et al. 2025), showed lineage-specific amino acid substitutions in both the substrate-binding and AAA domains (Fig. 6a2). *YME1L1*, the catalytic core of the i-AAA protease involved in OXPHOS maintenance (Puchades et al. 2017), was both a PSG and a HRERG, with lineage-specific substitutions located in its AAA and peptidase domains (Fig. 6a3). The matrix-localized CLPXP complex, which degrades misfolded proteins (Sauer et al. 2022), also exhibited derived features: whereas its core gene *CLPP* is typically single-copy in other butterflies, it underwent lineage-specific expansion in Miletinae, and the duplicated copies showed elevated sequence divergence on Miletinae branches in the gene tree (Fig. 6a4). *AFG3L2*, a central component of the m-AAA protease that degrades damaged or unassembled OXPHOS subunits (Puchades et al. 2019), also showed lineage-specific expansion together with elevated sequence divergence in Miletinae copies (Fig. 6a5).

#### Mitochondrial RNA degradation and stabilization

A comparable pattern was also observed in the system responsible for mitochondrial RNA degradation and stabilization. This system regulates the turnover and maintenance of mitochondrial transcripts and thereby contribute to mitochondrial RNA homeostasis (Santonoceto et al. 2024). Among the core genes associated with this system, four showed clear signatures of lineage-specific evolution in Miletinae (Fig. 6b1). *PNPT1*, a polynucleotide phosphorylase that forms the mitochondrial degradosome together with the RNA helicase *SUPV3L1* (Silva et al. 2018), was both a PSG and a HRERG, with lineage-specific amino acid substitutions located in its RNase_PH_C, PNPase, and RNase_PH domains (Fig. 6b2). *SUPV3L1*, the helicase component of the mitochondrial degradosome, was also identified as a PSG and a HRERG, and showed lineage-specific substitutions in its Helicase_C and Suv3_C_1 domains (Fig. 6b3). In addition to RNA degradation, genes involved in transcript stabilization also exhibited derived features. *LRPPRC*, a RNA-binding factor associated with mitochondrial mRNA stability and polyadenylation (Barchiesi and Vascotto 2019), underwent lineage-specific expansion in Miletinae, and its duplicated copies showed elevated sequence divergence on Miletinae branches in the gene tree (Fig. 6b4). A similar pattern was observed for *MTPAP*, the mitochondrial poly-A polymerase, which also showed lineage-specific expansion together with elevated sequence divergence in Miletinae copies (Fig. 6b5). Together, these findings show that compensatory evolution in the nuclear genome of Miletinae extended beyond direct mitonuclear contact sites to include broader maintenance systems required for mitochondrial macromolecular homeostasis. The coordinated evolution of protein quality control and mtRNA turnover systems indicates that the nuclear response to altered mitochondrial evolution in this lineage was not limited to restoring specific structural interfaces, but also involved broader remodeling of cellular systems associated with mitochondrial maintenance.

## Discussion

### Extreme ecological niche shifts may cause disruption of mitonuclear coadaptation

Our study suggests that extreme ecological niche shifts may provide a previously underappreciated route to disruption of mitonuclear coadaptation. Most empirical studies of mitonuclear incompatibility have focused on hybridization and introgression, in which mitochondrial and nuclear genomes with different evolutionary histories are brought together within a shared genetic background. Experimental and natural systems such as *Tigriopus*, *Nasonia*, and hybrid swordtails have shown that such mismatched combinations can reduce mitochondrial performance, disrupt OXPHOS, and contribute directly to hybrid breakdown (Ellison and Burton 2006; Niehuis et al. 2008; Burton and Barreto 2012; Barreto and Burton 2013a; Healy and Burton 2023; Moran et al. 2024). By contrast, our study points to a distinct but conceptually related scenario: mitonuclear mismatch may also arise without hybridization, when a lineage undergoes an extreme ecological transition. In Miletinae, the transition from the ancestral phytophagous condition of Lepidoptera to obligate carnivory represents an exceptional trophic shift (Pierce 1995), and our data indicate that this transition was accompanied by a marked breakdown of the ancestral balance between mitochondrial and nuclear genome evolution. Relative to phytophagous butterflies, Miletinae show a strong acceleration of mitochondrial sequence evolution but no comparable genome-wide acceleration in the nuclear genome, reversing the usual lepidopteran pattern in which nuclear coding sequences evolve faster than mitochondrial genes (Tao et al. 2024). This asymmetric response is consistent with the intrinsic evolutionary properties of the mitochondrial genome, including its lack of recombination (Ballard and Whitlock 2004), smaller effective population size (Moore 1995), high mutation rate (Lynch et al. 2006), and greater susceptibility to mutational erosion (Lynch and Blanchard 1998; Neiman and Taylor 2009). Under a major ecological perturbation, these properties may allow the mitochondrial genome to deviate more readily from the ancestral coadapted state than the nuclear genome, amplifying the intrinsic evolutionary asymmetry between the mitochondrial and nuclear genomes, thereby destabilizing previously coadapted mitonuclear combinations.

Interestingly, our results suggest that accelerated mitochondrial evolution in Miletinae was driven mainly by relaxation of ancestral selective constraints rather than by direct adaptive optimization. This pattern is notable because a major trophic transition might intuitively be expected to favor adaptive change in genes involved in energy metabolism. One plausible explanation is that, during or after the transition to carnivory, the selective regime maintaining the ancestral phytophagy-adapted mitonuclear genotype was weakened, allowing the mitochondrial genome to drift away from its previous coadapted state. Two nonmutually exclusive mechanisms may account for this relaxation. First, the shift from plant-based diets to prey-derived resources may have changed the metabolic demands imposed on mitochondrial pathways. Phytophagy requires coping with plant structural carbohydrates, nutrient imbalance, and diverse secondary metabolites, whereas carnivory provides animal protein- and lipid-rich resources and may alter reliance on particular mitochondrial substrates or OXPHOS modules (Yu et al. 2014; Putti et al. 2015; Aw et al. 2018). Such a transition could weaken the ancestral selective regime that maintained mitochondrial function in a phytophagous context, even if new selective pressures later arose under carnivory. Second, the origin of carnivory may have involved founder effects, demographic bottlenecks, or ecological specialization, reducing effective population size and thereby weakening the efficacy of purifying selection in the nonrecombining mitochondrial genome (Pierce 1995; Kaliszewska et al. 2015). These processes may help explain why mitochondrial evolution in Miletinae bears a stronger signature of relaxed constraint than of pervasive adaptive optimization.

Comparable patterns have also been observed in other lineages that underwent successful extreme ecological transitions, such as the shift from free-living to parasitic lifestyles in parasitic barnacles (Jung et al. 2025), from perennial to annual life history in African killifish (Cui et al. 2020), and from parasitic to predatory lifestyles in mites (Havird et al. 2026). In these lineages, the mitochondrial genome underwent accelerated evolution relative to close relatives, and the elevated evolutionary rates were largely driven by nonadaptive processes. In this view, the mitochondrial genome in Miletinae did not initially move toward a new optimum through pervasive adaptive selection; instead, it first experienced a loss of ancestral constraint, creating the potential for mitonuclear mismatch. The strong signatures of nuclear compensation that we detect are consistent with this interpretation and suggest that the long-term persistence of Miletinae after the shift to carnivory depended less on adaptive remodeling of mitochondrial genes themselves than on compensatory changes in the nuclear genome. We therefore propose a disruption–compensation model of mitonuclear evolution, in which extreme ecological transitions first relax the selective regime maintaining an ancestral mitonuclear genotype, allowing the mitochondrial genome to deviate from its previous coadapted state; subsequent multilayered nuclear compensation then enables the emergence of a new functional mitonuclear configuration. This framework suggests that successful ecological transitions may sometimes require not only adaptation to a new niche, but also restoration of functional coordination between mitochondrial and nuclear genomes after ancestral coadaptation has been perturbed.

It is important, however, to distinguish this disruption–compensation scenario from a general expectation that ecological niche shifts necessarily disrupt mitonuclear coadaptation. Mitonuclear interactions are often strongly context-dependent, and their fitness consequences can vary across environments rather than being uniformly deleterious (Sujkowski et al. 2019; Nguyen et al. 2020; Rodríguez et al. 2021; Nguyen et al. 2025). In *Caenorhabditis elegans*, for example, large-scale cybrid experiments showed that some mismatched mitonuclear combinations can perform better under specific environmental conditions, indicating that mitonuclear genotypes can retain a degree of ecological flexibility (Nguyen et al. 2025). Similarly, comparative work in ecologically diverse *Bombus* species suggests that mitonuclear coevolution can contribute to climatic niche adaptation and thermoregulation without necessarily leading to system-wide breakdown (Gonçalves et al. 2025). Therefore, disruption of mitonuclear coadaptation along with ecological niche shift is likely to be a relatively uncommon outcome, most likely to occur when ecological transitions are both extreme and imposed on lineages whose ancestral niche has remained strongly conserved over long evolutionary timescales. Under such conditions, the shift may exceed the buffering capacity of preexisting mitonuclear flexibility, thereby increasing the likelihood of incompatibility and subsequent compensatory evolution.

### Nuclear compensation after mitonuclear disruption: from direct interfaces to system-level remodeling

The classical nuclear compensatory framework proposes that because mitochondrial genomes are especially prone to mutational erosion, changes in mitochondrial genes can impose selective pressure on interacting nuclear genes, which then evolve in a compensatory manner to preserve mitochondrial function (Hill 2020). In this respect, our results provide strong support for the core prediction of the theory. In Miletinae, the clearest signatures of nuclear response are concentrated in the systems most directly coupled to mitochondrial function, especially OXPHOS complexes and the mitochondrial translation machinery. Nuclear genes encoding proteins that physically interact with mitochondrial gene products show elevated RERs and frequent signatures of positive selection, whereas appropriate controls do not. For example, nuOXPHOS genes track the accelerated evolution of mtOXPHOS genes, but glycolysis genes do not; similarly, MTRP and mtARS coevolve with mitochondrial RNAs, whereas their cytoplasmic counterparts do not. These contrasts indicate that the nuclear response in Miletinae was not a diffuse consequence of carnivory, but was preferentially concentrated in loci most likely to maintain compatibility with rapidly changing mitochondrial components.

In addition, our study broadens the empirical scope of nuclear compensatory evolution. Most previous work has emphasized classical protein–protein and protein–RNA interfaces, particularly OXPHOS complexes and the mitoribosome (Osada and Akashi 2012; Barreto and Burton 2013b; Barreto et al. 2018). Although our results strongly support the importance of these systems, they also show that compensatory responses in Miletinae extend beyond them. We detected strong lineage-specific signatures in *POLRMT*, *TFB2M*, and *TFAM*, key nuclear factors involved in mitochondrial transcription and replication (Gustafsson et al. 2016; Hillen et al. 2017; Hill 2019; Kozhukhar and Alexeyev 2023). This pattern is especially notable because the mitochondrial control region is enlarged in Miletinae, and the factors that recognize or regulate this region show some of the clearest evidence of adaptive change. These observations suggest that compensation in this lineage also involved protein–DNA interactions associated with promoter recognition, transcription initiation, and replication control. Thus, the consequences of altered mitochondrial evolution were not limited to interfaces that assemble respiratory chain complexes or decode mitochondrial transcripts, but also extended to the machinery that reads and regulates the mitochondrial genome itself. These findings expand the current view of mitonuclear compensation and highlight the importance of considering mtDNA regulatory interactions, in addition to the classical structural interfaces.

An equally important implication of our study is that nuclear compensation may also operate at the level of mitochondrial maintenance and homeostasis. Genes involved in mitochondrial protein quality control, RNA degradation, and transcript stabilization show repeated signals of lineage-specific expansion, accelerated evolution, or positive selection in Miletinae. This pattern suggests that mitonuclear mismatch may generate cascading downstream effects that extend well beyond the immediate site of incompatibility (Cline 2012; Giorgi et al. 2018; Rong et al. 2021). If altered mitochondrial proteins, RNAs, or regulatory elements reduce the efficiency of OXPHOS and mitochondrial translation, then the burden on the systems responsible for processing, stabilizing, assembling, and degrading mitochondrial macromolecules should also increase. In this context, the evolution of proteases, RNA surveillance factors, and transcript maintenance genes may represent an additional buffering layer that helps preserve mitochondrial function under persistent incompatibility pressure. From this perspective, nuclear compensatory evolution should not be regarded simply as a few isolated substitutions at core interaction interfaces, but rather as multilayered remodeling across an integrated mitochondrial network. Although this pattern is documented here in Miletinae, similar system-level compensatory responses may also have occurred in other animal lineages whenever sustained mitonuclear mismatch places persistent pressure on mitochondrial maintenance systems.

Although nuclear compensation is often regarded as a prominent mode of mitonuclear coevolution, empirical evidence for its prevalence remains mixed (Sloan et al. 2017). Compensatory coevolution has been documented in several systems, whereas other studies have found little evidence for strong or pervasive nuclear compensation despite clear evidence of mitonuclear coevolution (Barreto and Burton 2013b; Barreto et al. 2018; Piccinini et al. 2021; Weaver et al. 2022; Zhao et al. 2025). Our results provide a possible framework for reconciling these conflicting observations. In Miletinae, mitochondrial evolution was accelerated mainly through relaxation of ancestral selective constraints rather than through adaptive optimization, implying that the ancestral mitonuclear state was destabilized before the emergence of nuclear compensatory responses. This suggests that nuclear compensation may not be a universal or constantly visible feature of mitonuclear evolution; instead, it may only become pronounced when preexisting coadaptation is disrupted, such as after hybridization, introgression, or, as we propose here, extreme ecological niche shift (Barreto and Burton 2013b; Barreto et al. 2018; Shi et al. 2025). In more stable evolutionary contexts, strong purifying selection may be sufficient to preserve compatible mitonuclear combinations, making extensive nuclear compensation unnecessary. In contrast, when a lineage experiences a transition severe enough to destabilize the ancestral mitonuclear state, nuclear compensation may act as a rescue mechanism that permits survival and longer-term persistence. Miletinae may represent precisely such a case. Their success as the only major carnivorous butterfly lineage suggests that extreme trophic transitions in Lepidoptera may be constrained not only by ecological opportunity, but also by the need to overcome a deep compatibility barrier between mitochondrial and nuclear genomes. More generally, our study links ecological niche evolution with the dynamics of mitonuclear coevolution and supports a model in which extreme niche shifts can first destabilize ancestral mitonuclear genotypes and then promote the emergence of a new coadapted state through multilayered nuclear compensation.

## Materials and methods

### Sample collection, DNA/RNA extraction, and sequencing

Adult individuals of *T. hamada* and *M. chinensis* were collected from Yunnan, China, and one adult *A. drumila* individual was collected from Guangzhou, China. To reduce contamination from gut-associated microbes, guts were removed from freshly collected specimens, and the remaining tissues were washed twice with PBS buffer. Dry-pinned specimens of *M. mallus*, *S. epius*, and *T. shiloi* were obtained from the Insect Collections at the College of Agronomy, South China Agricultural University. Genomic DNA of freshly collected specimens was extracted from whole-body tissues (without guts) using the Magnetic Animal Tissue Genomic DNA Kit (TIANGEN, Beijing, China). For dry-pinned specimens, DNA was extracted from thoracic tissue or legs. Total RNA was extracted from one *T. hamada* individual using TRIzol Reagent (Invitrogen, USA). DNA and RNA quality were evaluated using a Qubit 3.0 Fluorometer and 1.0% TBE agarose gel electrophoresis.

For nuclear genome sequencing, PacBio HiFi libraries were prepared for *T. hamada*, *M. chinensis*, and *A. drumila*. Two 20-kb SMRTbell libraries were constructed for *T. hamada* and *M. chinensis*, respectively, whereas a 10-kb library was constructed for *A. drumila* because of limited DNA quantity. Hi-C libraries were generated for *T. hamada* and *M. chinensis* and sequenced on the Illumina NovaSeq 6000 platform with 150-bp paired-end reads. RNA-seq libraries for *T. hamada* were prepared using the Illumina TruSeq Stranded mRNA Library Prep Kit (Illumina Inc., USA) and sequenced on the NovaSeq 6000 platform. For *M. mallus*, *S. epius*, and *T. shiloi*, mitochondrial DNA was enriched using Lepidoptera-specific mtDNA capture baits following Zhu et al. (2025), and enriched libraries were sequenced on the Illumina NovaSeq 6000 platform in PE150 mode.

### Genome assembly and annotation

Genome size and heterozygosity of *T. hamada* were estimated from Illumina short reads using Jellyfish v2.3.0 (Marçais and Kingsford 2011) and GenomeScope v1.0 (Vurture et al. 2017). The genomes of *T. hamada*, *M. chinensis*, and *A. drumila* were assembled with Hifiasm v0.19.6 (Cheng et al. 2021). Haplotigs and heterozygous overlaps were removed using purge_dups v1.2.6 (Guan et al. 2020), and potential contaminant sequences were filtered using FCS-GX v0.5.4 (Astashyn et al. 2024). Chromosome-scale assemblies of *T. hamada* and *M. chinensis* were generated using Hi-C data. Hi-C reads were processed with Juicer v1.6.2 (Durand et al. 2016b), and contigs were ordered and oriented with 3D-DNA v190716 (Dudchenko et al. 2017). Hi-C contact maps were inspected and manually corrected in Juicebox v2.17.0 (Durand et al. 2016a). Telomeric repeats were identified using quarTeT v1.2.5 (Lin et al. 2023). Assembly completeness was assessed using BUSCO v5.4.3 (Manni et al. 2021) with the Lepidoptera_odb10 database.

Repetitive elements were annotated by integrating homology-based and de novo prediction strategies. Specifically, a de novo repeat library was first constructed from the assembled genomes using RepeatModeler v2.0.3 (Flynn et al. 2020). This library was merged with known repeat element databases, including the Insecta subset of Repbase-20181026 (Bao et al. 2015) and Dfam 3.7 (Hubley et al. 2016), to generate a custom repeat library. RepeatMasker v4.1.5 (Tarailo-Graovac and Chen 2009) was then used to identify and mask repetitive regions in the genome assemblies.

Protein-coding genes were predicted by integrating ab initio, homology-based, and transcriptome-based (when available) approaches. In brief, Braker3 v3.0.7 (Gabriel et al. 2023), Helixer v0.3.4 (Stiehler et al. 2021), and SNAP v2006-07-28 (Korf 2004) were used for ab initio gene prediction. For homology-based prediction, protein sequences from six species with high-quality gene sets were used to construct a homology reference dataset. Gene structures were predicted with GeMoMa v1.9 (Keilwagen et al. 2016), GenomeThreader v1.7.3 (Gremme et al. 2005), and Miniprot v0.12 (Li 2023). RNA-seq reads were aligned to the genomes with HISAT2 v2.2.1 (Kim et al. 2015), and genome-guided transcript assembly was performed with StringTie v2.2.1 (Pertea et al. 2015). Open reading frames within assembled transcripts were identified using TransDecoder v5.5.0. Gene models from the 3 approaches were integrated with EvidenceModeler v2.1.0 (Haas et al. 2008) to produce high-quality gene sets. Functional annotation of predicted protein-coding genes was carried out with eggNOG-mapper v2.1.12 (Huerta-Cepas et al. 2017) against the eggNOG v5.0 database (Huerta-Cepas et al. 2019). Protein domains were predicted using InterProScan v5.59-91.0 (Finn et al. 2017). Mitonuclear genes were identified using the MitoCarta database and assigned to their respective mitochondrial pathways (Rath et al. 2021). Completeness of the final gene sets was evaluated with BUSCO v5.4.3 using the Lepidoptera_odb10 database.

Mitochondrial genomes of *A. drumila*, *M. chinensis*, and *T. hamada* were assembled from HiFi reads using MitoHiFi v3.2.2 (Uliano-Silva et al. 2023). The mitochondrial genomes of *M. mallus*, *S. epius*, and *T. shiloi* were assembled from the captured data using GetOrganelle v1.7.5 (Jin et al. 2020) and MetaSPAdes v3.13.0 (Nurk et al. 2017). All mitochondrial genomes used in this study were annotated with MITOS v2.1.9 (Bernt et al. 2013).

### Phylogenomic analyses

Genome assemblies of 45 butterfly species (7 families and 45 genera) and 3 moth species (3 families) were downloaded from the National Center for Biotechnology Information (NCBI) and the Darwin Tree of Life project (DToL) (Table S7). These genomes were selected based on three criteria: (i) to ensure phylogenetic representativeness, species from different tribes or genera were sampled within each family whenever possible; (ii) high completeness (BUSCO completeness >90%); and (iii) high contiguity (contig N50 > 5 kb and scaffold N50 > 500 kb). For 34 genomes lacking annotated gene sets, gene annotation was performed using the same pipeline described above, and the longest transcript per gene was retained. BUSCO v5.4.3 with the Lepidoptera_odb10 database was used to extract the protein and CDS sequences of nuclear BUSCO (nuBUSCO) genes from the genomes. Protein sequences of each nuBUSCO gene were aligned using MAFFT v7.505 (Katoh and Standley 2013). Codon alignments were generated based on the protein alignments using custom scripts, and poorly aligned regions were trimmed with Gblocks v0.91b (Castresana 2000), employing the relaxed filtering parameters -b4 = 5 -b5 = h. The 13 mtPCGs were aligned following the same procedure. For both protein and codon datasets, only alignments with less than 20% missing taxa were retained for downstream phylogenetic analyses.

The nuclear ML phylogeny was reconstructed from the concatenated protein alignment of 5,150 nuBUSCO genes using IQ-TREE v2.2.2.7 (Minh et al. 2020) under the LG + G4 model. The mitochondrial ML phylogeny was reconstructed from the concatenated protein alignment of the 13 mtPCGs under the mtInv + F + G4 model. Node support for both phylogenies was assessed using 5,000 ultrafast bootstrap replicates (-bb 5000). To evaluate whether the nuclear phylogeny was sensitive to substitution model or partitioning strategy, we additionally performed partitioned ML analyses. Following Wang et al. (2020), evolutionary rates were estimated for each of the 5,150 nuBUSCO genes, and genes with similar rates were grouped into 250 partitions of comparable size. The best-fitting substitution model for each partition was selected using ModelFinder (Kalyaanamoorthy et al. 2017) implemented in IQ-TREE (-m MFP). The partitioned analysis recovered a topology identical to that obtained from the unpartitioned analysis, indicating that the inferred nuclear phylogeny was robust to partitioning strategy and model specification. Following previous studies of insect mitonuclear ERC (Tao et al. 2024), we then re-estimated mitochondrial branch lengths on a common nuclear phylogenetic framework. Specifically, branch lengths were estimated from the concatenated mtPCG protein alignment with the topology constrained to the nuBUSCO tree, using the mtInv + F + G4 model. This constrained analysis allowed mitochondrial and nuclear evolutionary patterns to be compared on the same topology.

For substitution-rate estimation, nucleotide ML trees were inferred from concatenated CDS alignments of the nuBUSCO genes and mtPCGs under the GTR + G4 model. Species-specific nucleotide substitution rates for the nuclear and mitochondrial genomes were then estimated using the penalized-likelihood approach implemented in r8s v1.81 (Sanderson 2003), based on ML trees inferred from concatenated CDS alignments and calibrated with divergence times retrieved from the TimeTree database (Kumar et al. 2017). The divergence between *Helicoverpa zea* and *Papilio machaon* was constrained to 55.8 to 140.4 Ma, whereas the divergences between *P. machaon* and *Danaus plexippus* and between *Apodemia mormo* and *Aricia agestis* were set to 97 Ma and 88 Ma, respectively.

### Mitochondrial genome evolution analyses

Branch lengths for each mitochondrial gene were estimated using IQ-TREE v2.2.2.7 under the best-fit substitution model, with the tree topology constrained to the nuclear phylogeny. The 22 mitochondrial tRNA genes were concatenated and analyzed as a single partition. For each mitochondrial gene, the evolutionary rate was measured as the root-to-tip branch length. Overall mitochondrial *dN/dS* ratios for each species were estimated from the concatenated 13 mtPCG alignment using the free-ratio model (model = 1, NSsites = 0, fix_omega = 0, icode = 4) in CodeML implemented in PAML v4.10.6 (Yang 2007). To elucidate the pattern of selection acting during the evolutionary transition to carnivory, RELAX was implemented in HyPhy v2.5.63 (Kosakovsky Pond et al. 2020) under the minimal model. RELAX analyses were performed for both individual mtPCGs and the concatenated mtPCG matrix. The model estimates *dN/dS* variation across sites using 3 discrete rate categories, and significance was assessed using LRTs.

PGLS analysis was used to test whether the association between diet and mitochondrial genome substitution rate remained significant after accounting for phylogenetic relatedness. The analysis was performed using the *gls* function in the nlme package v3.1-164 (Pinheiro et al. 2023), based on the mitochondrial tree. Mitochondrial genome substitution rate was specified as the response variable and diet (phytophagy = 0, carnivory = 1) as the explanatory variable. Regression coefficients, *t*-statistics, and corresponding *P*-values were used to evaluate the association between diet and substitution rate.

To determine whether the increase in mitochondrial substitution rate occurred during the dietary transition itself or during the subsequent evolution of carnivorous lineages, branch-specific evolutionary rate changes were estimated using the RRphylo function in the RRphylo package v3.0.2 (Castiglione et al. 2018). Significant rate-shift branches were identified using the search.shift function. Branches with empirical probabilities greater than 0.975 were interpreted as showing significantly higher rates than the background, whereas those with empirical probabilities lower than 0.025 were interpreted as showing significantly lower rates. Branch-specific rate-change estimates were then extracted for the branch corresponding to the phytophagy–carnivory shift and for descendant carnivorous branches, and the magnitude of rate acceleration between these two branch categories was compared using a permutation test.

### Chromosomal evolution analysis

Lepidopteran ancestral linkage groups (ALGs), also referred to as Merian elements, were identified for eight lycaenid species representing four subfamilies (Lycaeninae, Miletinae, Polyommatinae, and Theclinae), following Wright et al (2024). The Ri between each species' genome and the Merian elements was calculated following Lewin et al. (2024). Ancestral Ri values were inferred using phytools v2.4.4 (Revell 2024), based on divergence times and phylogenetic relationships. Because extensive chromosomal rearrangements were detected in the genomes of *M. chinensis* and *T. hamada*, fusion and fission events were inferred using 2 complementary approaches: syngraph (Mackintosh et al. 2023) and lep_fusion_fission_finder (LFFF; available at https://github.com/charlottewright/lep_fusion_fission_finder). Following Wright et al. (2024), a threshold of 17 orthologues was applied for both syngraph and LFFF, which produced nearly identical results. Syngraph was used to infer chromosome numbers at each internal node of the phylogeny and to identify branch-specific fusion and fission events. LFFF was used to detect fusion and fission events at internal nodes and to determine the predominant Merian elements associated with each chromosome, thereby characterizing chromosome rearrangement states.

### Gene family evolution analysis

For gene family evolution analysis, 20 high-quality genomes (including 3 Miletinae species and 17 outgroup species) were selected from the phylogenomic datasets based on two criteria: BUSCO completeness >95% and high assembly contiguity based on PacBio HiFi or Oxford Nanopore ultra-long-read data (Table S7). Orthologous groups (OGs) were inferred among the 20 species using OrthoFinder v2.5.4 (Emms and Kelly 2019), based on the longest transcript of each gene. Divergence times were estimated using MCMCTREE in PAML v4.10.6, calibrated with divergence times retrieved from the TimeTree database described above. Gene family expansions and contractions were inferred using CAFÉ v5.0 (Mendes et al. 2021), with OrthoFinder-derived gene families as input. Gene families with *P* < 0.05 were considered significantly expanded or contracted. For each gene family, *dN/dS* ratios in the Miletinae clade were estimated using the branch model in CodeML in PAML v4.10.6 (model = 2, NSsites = 0), and significance was assessed by LRTs against the one-ratio null model (model = 0, NSsites = 0).

### Positive selection analysis

For positive selection analysis, single-copy OGs among the 20 species were identified using SonicParanoid v2.0.8 (Cosentino et al. 2024). Protein sequences for each single-copy OG were aligned with MAFFT v7.505, codon alignments were generated from protein alignments, and poorly aligned regions were trimmed with Gblocks v0.91b. A total of 7,215 single-copy OGs containing all three Miletinae species and at least 10 outgroup species were retained. PSGs were identified using the branch-site model (model = 2, NSsites = 2, fix_omega = 0) implemented in CodeML in PAML v4.10.6, with the ancestral branch of Miletinae specified as the foreground branch. Model fit was assessed by comparing the alternative model with the null model (model = 2, NSsites = 2, fix_omega = 1, ω = 1) using LRTs, followed by Benjamini–Hochberg (BH) false discovery rate correction. Single-copy OGs with an adjusted *P* < 0.05 and at least one positively selected site with Bayes Empirical Bayes (BEB) posterior probability >0.95 were considered PSGs. The same branch-site framework was applied to individual mitochondrial genes along the ancestral branch of Miletinae. GO enrichment analysis of PSGs was performed using clusterProfiler v4.10.0 (Wu et al. 2021).

### Protein RER analysis

The 7,215 single-copy OGs used for positive selection analysis were also used to identify HRERGs, following Richardson et al. (2023). Briefly, branch lengths for the protein sequence of each single-copy OG were estimated with the species-tree topology fixed. RERconverge v0.3.0 (Redlich et al. 2024) was then used to scale each locus to the master tree, and residual branch lengths are taken as the RER for each single-copy OG. Genes with significantly elevated RERs on the Miletinae branch relative to the genomic background (*P* < 0.05) were identified as HRERGs. Functional enrichment analyses of HRERGs were performed using Metascape (Zhou et al. 2019), and protein–protein association networks for HRERGs were constructed using the STRING database (Szklarczyk et al. 2023).

### Conserved noncoding element analysis

Whole-genome alignments (WGAs) of 3 Miletinae species and 17 outgroup species were generated using Progressive Cactus v2.9.3 (Armstrong et al. 2020), with the species tree used as the guide tree. The resulting HAL file was converted to a nonredundant MAF file using cactus-hal2maf with the options --noAncestors --filterGapCausingDupes --dupeMode single, using *M. chinensis* as the reference genome. A neutral substitution model was estimated from 4-fold degenerate sites using phyloFit in PHAST v1.5 (Hubisz et al. 2011), with the options subst-mod = REV, precision = HIGH. Evolutionarily conserved regions across the 20 species were identified with phastCons in PHAST using this neutral model, with the options target coverage = 0.3, expected length = 45, and rho = 0.3. CNEs were obtained by removing exonic regions from the identified conserved regions using BEDtools v2.30.0 (Quinlan and Hall 2010), and only CNEs longer than 30 bp were retained. We used PhyloAcc-GT v2.4.2 (Yan et al. 2023) to infer rate-shift states of CNEs across species and considered CNEs assigned to model M1 (BF1 ≥ 10 and BF2 ≥ 1) as Miletinae-specific aCNEs in the Miletinae clade. Regulatory ranges for each aCNE were defined following McLean et al. (McLean et al. 2010), including a basal regulatory range extending 5-kb upstream and 1-kb downstream of the transcription start site (TSS) and an extended regulatory range of up to 1 Mb in both directions. Each aCNE was assigned to its nearest gene using the annotatePeaks module in HOMER v5.1 (Heinz et al. 2010). GO enrichment analysis of genes neighboring aCNEs was performed using clusterProfiler v4.10.0.

### ERC analysis

Gene sets used for ERC analysis were obtained from FlyBase (Öztürk-Çolak et al. 2024), including nuOXPHOS (76 nuclear-encoded OXPHOS proteins), nuOXPHOS CII (4 nuclear-encoded complex II OXPHOS proteins), nuOXPHOS without CII (72 nuclear-encoded OXPHOS proteins), MTRP (75 mitochondrial ribosomal proteins), CRP (93 cytoplasmic ribosomal proteins), mtARS (16 mitochondrial aminoacyl-tRNA synthetases), nuARS (16 cytoplasmic aminoacyl-tRNA synthetases), and glycolysis (12 glycolytic genes) (Table S15). SonicParanoid v2.0.8 (Cosentino et al. 2024) was used to identify homologous sequences for each gene in these sets across 51 species. Each gene was aligned, trimmed, manually inspected, and concatenated into protein and CDS supergene matrices. The mtOXPHOS gene set, comprising 13 mitochondrial-encoded OXPHOS proteins, and the mtRNA gene set, comprising 24 mitochondrial RNA genes, were obtained from the mitochondrial genome analyses described above.

ERC analyses were performed by integrating the approaches of Weaver et al. (Weaver et al. 2022) and Tao et al. (Tao et al. 2024). Branch lengths were estimated for each single-gene alignment and each supergene matrix with the species-tree topology fixed. The three moth species were used as outgroups for branch-length estimation but were excluded from downstream ERC analyses. RERs were calculated by dividing branch lengths for each gene or supergene matrix by the corresponding branch lengths of the species tree. Because mtOXPHOS and mtRNA showed exceptionally high RER in Miletinae, Spearman's rank correlation was used to assess rate covariation between mtOXPHOS or mtRNA and each nuclear gene set. We generated the distribution of Spearman correlation coefficients (*r_s_*) for each comparison by performing 10,000 bootstrap resamplings to evaluate differences in ERC strength. To examine whether mitonuclear coevolution holds across butterfly lineages and to assess the influence of Miletinae, we repeated the same analyses after removing Miletinae species.

### Protein structure analysis

Protein structural models were downloaded from the PDB database (Burley et al. 2025) to visualize and assess physical contacts among subunits, including OXPHOS complex I (5LNK), complex II (8GS8), complex III (8P65), complex IV (1V54), complex V (8H9V), mitoribosome (3J9M), mt-SerRS (7U2B), and the mitochondrial transcription initiation complex (6ERQ). Physical interactions among subunits were identified using the “identify contacts/clashes” tool in Chimera v1.19 (Pettersen et al. 2004), with the overlap parameter set to ≤−1 Å to increase sensitivity.

## Supplementary Material

msag221_Supplementary_Data

## Data Availability

All raw sequencing data and the genome assemblies underlying this article are available at the NCBI and can be accessed with Bioproject ID: PRJNA1441805 (reviewer link: https://dataview.ncbi.nlm.nih.gov/object/PRJNA1441805?reviewer=rgurlnsidt6fgmd10bkrmqfus7). Soft-masked genome files and genome annotation files are available on Figshare under DOI: 10.6084/m9.figshare.32089197.
