# Supplementary material for: Disruption of mitonuclear coadaptation and compensatory evolution after an extreme dietary shift in carnivorous butterflies
Source: Mol Biol Evol. 2026 Aug 27;43(9):msag221. doi: 10.1093/molbev/msag221 (PMC13560083; doi:10.1093/molbev/msag221)
Supplement: Supplementary file 1 [file msag221_supplementary_data.zip › Revised Supplementary Figure 1-9 final.docx]

**Figure S1.** Genome survey of *Taraka hamada*. GenomeScope profile was estimated from Illumina short-read k-mer frequencies. The model estimated a genome size of approximately 456.7 Mb, with 0.578% heterozygosity.

**Figure S2.** Hi-C contact maps of the chromosome-scale genome assemblies of *Taraka hamada* and *Miletus chinensis*.

**Figure S3** Gene density and telomere recovery across the chromosome-scale assemblies of *Taraka hamada* and *Miletus chinensis*.

**Figure S4** Genome synteny among eight chromosome-level lycaenid genomes. Gray links indicate conserved syntenic blocks between genomes. Extensive interchromosomal syntenic connections in Miletinae (red chromosomes) indicate substantial chromosomal rearrangement relative to other lycaenid lineages.

**Figure S5.** Distinct mitochondrial and nuclear substitution-rate patterns between Miletinae and phytophagous butterflies. (**a**) Comparison of mitochondrial nucleotide substitution rates. (**b**) Comparison of nuclear nucleotide substitution rates. (**c**) Comparison of nuclear and mitochondrial nucleotide substitution rates within phytophagous butterflies. (**d**) Comparison of nuclear and mitochondrial nucleotide substitution rates within Miletinae. P-value were calculated using the Mann–Whitney U test.

**Figure S6.** Branch-specific changes in mitochondrial substitution rates across the Lepidoptera phylogeny. Phylogenetic branches are color-coded according to the rate of change in mitochondrial substitution rate. Circles at internal nodes indicate significant rate shifts, with red representing significant increases and blue representing significant decreases. Circle size is proportional to the magnitude of the rate difference relative to the background branches.

**Figure S7.** Hypergeometric tests were used to assess enrichment of mitonuclear genes among (a) high relative evolutionary rate genes (HRERGs), (b) positively selected genes (PSGs), and (c) genes associated with accelerated conserved noncoding elements (aCNEs). Orange bars indicate the observed proportions of mitonuclear genes in each candidate gene set, and gray bars indicate the genomic background. P values and fold-enrichment values are shown above each comparison.

**Figure S8.** Metascape enrichment network and bar plot showing the top 20 ontology clusters enriched among the 334 high relative evolutionary rate genes. Nodes represent enriched terms, and edges connect related terms. The bar plot shows the significance of representative enriched clusters, measured as -log10(P).

**Figure S9.** Comparison of mitochondrial genome size between Miletinae and phytophagous butterflies.

Miletinae show significantly larger mitochondrial genomes than phytophagous butterflies. P value was calculated using the Mann-Whitney U test.
